# Hypomorphic function and somatic reversion of DOCK8 cause combined immunodeficiency without hyper-IgE

**DOI:** 10.1016/j.clim.2015.12.003

**Published:** 2016-02

**Authors:** Anne-Kathrin Kienzler, Pauline A. van Schouwenburg, John Taylor, Ishita Marwah, Richa U. Sharma, Charlotte Noakes, Kate Thomson, Ross Sadler, Shelley Segal, Berne Ferry, Jenny C. Taylor, Edward Blair, Helen Chapel, Smita Y. Patel

**Affiliations:** a Nuffield Department of Medicine, Experimental Medicine Division, University of Oxford, UK; b Oxford NIHR Biomedical Research Centre, John Radcliffe Hospital, Oxford, UK; c Oxford NHS Regional Molecular Genetics Laboratory, Oxford University Hospitals NHS Trust, Oxford, UK; d Department of Clinical Laboratory Immunology, Churchill Hospital, Oxford University Hospitals NHS Trust, Oxford, UK; e Department of Paediatrics, Children's Hospital, Oxford University NHS Hospitals Trust, Oxford, UK; f Oxford Biomedical Research Centre, Wellcome Trust Centre for Human Genetics, Oxford, UK; g Department of Clinical Genetics, Churchill Hospital, Oxford University NHS Hospitals Trust, Oxford, UK

**Keywords:** CFSE, Carboxyfluorescein diacetate, succinimidyl ester, DHR1/2, DOCK homology region, DOCK8, Dedicator of cytokinesis 8, EBV, Epstein–Barr-Virus, HC, Healthy control, Mut, Mutated DOCK8 transcript (referring to c.6019dupT), PBMC, Peripheral blood mononuclear cell, PHA, Phytohemagglutinin, Pt, Patient, Trunc, Truncated DOCK8 protein, DOCK8, Combined immunodeficiency, Whole exome sequencing, Hyper-IgE syndrome, Phenotypic variability

## Abstract

Loss-of-function mutations in DOCK8 are linked to hyper-IgE syndrome. Patients typically present with recurrent sinopulmonary infections, severe cutaneous viral infections, food allergies and elevated serum IgE. Although patients may present with a spectrum of disease-related symptoms, molecular mechanisms explaining phenotypic variability in patients are poorly defined. Here we characterized a novel compound heterozygous mutation in *DOCK8* in a patient diagnosed with primary combined immunodeficiency which was not typical of classical DOCK8 deficiency. In contrast to previously identified mutations in *DOCK8* which result in complete loss of function, the newly identified single nucleotide insertion results in expression of a truncated DOCK8 protein. Functional evaluation of the truncated DOCK8 protein revealed its hypomorphic function. In addition we found somatic reversion of *DOCK8* predominantly in T cells. The combination of somatic reversion and hypomorphic DOCK8 function explains the milder and atypical phenotype of the patient and further broadens the spectrum of DOCK8-associated disease.

## Highlights

• Whole exome sequencing identified the underlying defect in a patient with combined immunodeficiency.
• A novel compound heterozygous DOCK8 mutation was identified.
• Expression of a truncated DOCK8 protein with hypomorphic function was identified.
• Somatic reversion of *DOCK8* mainly in T cells was identified.
• DOCK8 deficiency may present without severe viral infections and increased serum IgE levels.

## 1 Introduction

Bi-allelic loss-of-function mutations in the guanine-nucleotide exchange factor dedicator of cytokinesis 8 (*DOCK8*) cause autosomal recessive hyper-IgE syndrome. The vast majority of DOCK8-deficient patients present with combined immunodeficiency characterized by recurrent sino-pulmonary and/or gastrointestinal infections, severe cutaneous viral infections, severe atopy, eosinophilia and massively elevated serum IgE levels. Patients also have a predisposition to cancer [1], [2].

Recent studies have highlighted the phenotypic variability of patients suffering from DOCK8-deficiency [3], [4]. Patients with susceptibility to infection but less severe allergic disease were identified to carry a functional wild-type *DOCK8* allele in lymphocyte subpopulations due to somatic reversion of the mutated *DOCK8* alleles [3].

Here we report for the first time a patient with a hypomorphic mutation in *DOCK8* presenting with recurrent bacterial infections, low serum IgM and IgG, CD4 lymphopenia and severely impaired vaccination responses, but without severe viral infections and severe atopy.

## 2 Methods

Detailed information can be found in the Supplementary data.

We submitted the variants identified in DOCK8 to be made publically available by ClinVar (http://www.ncbi.nlm.nih.gov/clinvar/). The accession numbers are SCV000257461 (deletion chr9:204193-343954), SCV000257462 (c.65C>T), SCV000257463 (c.289C>A), SCV000257464 (c4107C>G), SCV000257465 (c.5433G>A), and SCV000257466 (c.6019dupT).

## 3 Case presentation

The female patient is the only child of non-consanguineous, healthy parents. She presented aged eight with a two-year history of recurrent bacterial chest infections and radiological signs of early bronchiectasis. The patient also had a long-standing history of mild eczema and asthma requiring treatment with inhaled corticosteroids and beta-agonists. All routine childhood immunizations were received uneventfully. Immunological evaluation (Table 1) revealed low serum IgM, normal IgA and IgE, and borderline-low IgG levels which dropped significantly over 12 months. Measurement of responses to previous immunizations demonstrated protective levels of IgG to tetanus toxoid but absent IgG to *Haemophilus influenzae type b*, pneumococcal polysaccharides and measles. Also, despite a history of a normal course of chicken-pox, varicella zoster virus IgG was undetectable. Lymphocyte subset analysis demonstrated low CD4^+^ T cell numbers and low frequencies of CD27^+^ effector B cells (Table 1). Following the failure of antibiotic prophylaxis alone to reduce the infection burden, immunoglobulin replacement therapy was commenced with a good clinical response. Sequence analysis of recombination-activating gene (*RAG*) *1*, *RAG2*, and DNA cross-link repair 1C (*DCLRE1C* encoding Artemis) did not reveal any mutations. Therefore the patient was given a diagnosis of undefined primary combined immunodeficiency.

## 4 Results and discussion

**Fig. 1:**
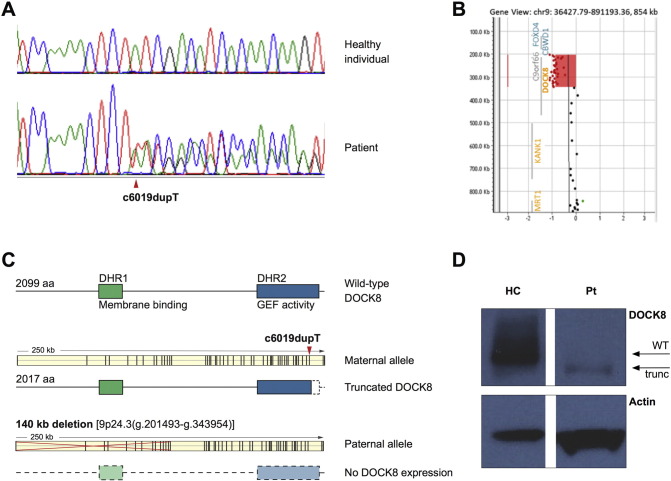
A novel compound heterozygous mutation in *DOCK8* results in expression of a truncated DOCK8 protein. (A) Sanger sequencing results for the single nucleotide duplication, c.6019dupT, p.(Tyr2007Leufs*12). The upper panel illustrates a normal control trace and the lower panel shows the presence of the mutation; the duplicated T nucleotide is indicated by the arrow. (B) Results of array comparative genomic hybridization illustrating the about 140 kb deletion in 9p24.3 (204,193–343,954). The deletion encompasses exons 1–14 of *DOCK8*. (C) Graphic depicting the wild-type DOCK8 protein structure and the outcome of the single-nucleotide insertion on the maternal allele and the deletion in *DOCK8* on the paternal allele on DOCK8 protein expression (*DOCK8* transcript reference is ENST00000453981). (D) DOCK8 protein expression in EBV-transformed B cells of a healthy control (7.5 μg protein lysate) and the patient (30 μg protein lysate). Actin was used as loading control.

To identify the underlying disease cause, we undertook whole exome sequencing (WES) on the patient and both parents. A novel heterozygous frameshift variant was detected in *DOCK8* in the patient and her mother. Sanger sequencing confirmed a single-nucleotide duplication [c.6019dupT (p.Tyr2007Leufs*12)] within the conserved DOCK homology region 2 (DHR2) domain of *DOCK8*, leading to a frameshift and premature stop-codon (Fig. 1, A and C, and Supplementary Table 1]. As autosomal recessive mutations in *DOCK8* cause combined immunodeficiency, we screened for further variants in *DOCK8*. Analysis of single nucleotide polymorphisms (SNPs) across *DOCK8* in the trio revealed apparent loss of paternal contribution of two SNPs in a 5′ region of the gene (Supplementary Table 1), indicating the possibility of a paternally inherited deletion. Array comparative genomic hybridization analysis confirmed a large deletion encompassing exons 1–14 of *DOCK8* in the patient and her father (approx. 140 kb deletion of 9p24.3, base pair 204,193 to 343,954) (Fig. 1, B and C). This novel compound heterozygous mutation in *DOCK8* was the only disease-causing variant identified in the patient (Supplementary Tables 2–4).

The deletion in *DOCK8* is predicted to result in the absence of any protein expression since the deletion includes the start codon. The frameshift mutation is predicted to result in the production of a truncated protein lacking 81 amino acids (~ 11 kDa). Indeed, patient EBV cells expressed low amounts of a truncated DOCK8 protein, but not the full-length protein (Fig. 1D). We hypothesized that this truncated DOCK8 protein has hypomorphic function accounting for the milder clinical presentation of our patient.

**Fig. 2:**
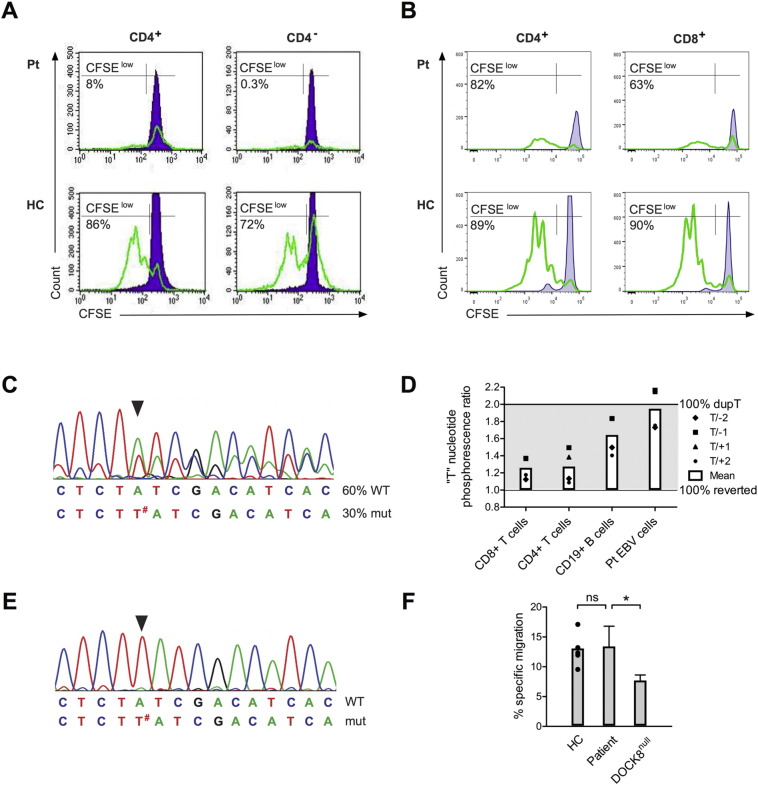
Improvement of T cell proliferation over time, somatic reversion of *DOCK8* in T cells and hypomorphic function of the truncated DOCK8 protein. Proliferation of PHA-stimulated, CFSE-labeled PBMCs of the patient at (A) 10 and (B) 15 years of age, and a healthy control. Depicted are percentages of CFSE^low^ cells gated on CD3^+^ CD4^+^ or CD4^−^ or CD8^+^ T cells. (C) Sanger sequence trace showing somatic reversion of the single nucleotide duplication (c.6019dupT) resulting in expression of about 60% wild-type *DOCK8* transcripts in the patient's CD3^+^ T cells. (D) “T” nucleotide phosphorescence ratios obtained by pyrosequencing *DOCK8* of primary CD3^+^ CD4^+^ and CD3^+^ CD8^+^ T cells, primary CD19^+^ B cells and the EBV B cell line of the patient. (T/± 1 or 2) depicts the signal ratio of c.6018-19T to nucleotides 1 and 2 positions up and downstream. The PCR templates and pyrosequencing reactions were performed in triplicate. Each symbol represents the mean of the three ratio measurements at respective nucleotide positions. The bar represents the mean “T” nucleotide phosphorescence ratio of all 4 different nucleotide ratios in indicated cell populations. (E) Sanger sequence trace showing expression of solely mutated *DOCK8* transcripts in EBV-transformed B cells of the patient. The duplicated T-nucleotide is indicated by the arrow and #. (F) Migration of EBV-transformed B cells of 5 different healthy controls (each symbol represents the mean of 3 independent experiments for each of the healthy control samples), the patient and a patient with a complete loss-of-function mutation in *DOCK8* (DOCK8^null^). The bar of the healthy control samples represents the mean of the mean of each of the 5 healthy control samples. The bar for each of the patient samples represents mean and standard deviation of 3 independent experiments for each sample.

Previous studies of DOCK8-deficient patients report impaired T cell proliferation [1], [2]. At the age of ten years, both CD4^+^ and CD8^+^ patient T cells did not proliferate in response to mitogen (PHA) stimulation (Fig. 2A), consistent with an inability of the truncated DOCK8 protein to transmit proliferative signals. Interestingly, when studying T cell proliferation at the time of WES (four years later), proliferation of both CD4^+^ and CD8^+^ patient T cells was present, although reduced compared to a healthy control (Fig. 2B). We hypothesized that this difference in T cell proliferation could be explained by somatic reversion of *DOCK8*. Indeed, Sanger sequencing of *DOCK8* cDNA of T cells and subsequent peak height quantification revealed that two thirds of all *DOCK8* transcripts are wild-type (Fig. 2C), showing somatic reversion of *DOCK8* as previously described [3]. Somatic reversion of *DOCK8* in T cells was confirmed by pyrosequencing of *DOCK8* (Fig. 2D). Therefore improved T cell proliferation over time is likely to be due to somatic reversion of *DOCK8* in patient T cells.

The frequency of somatic reversion in B cells was half compared to the *DOCK8* reversion in T cells (Fig. 2D) indicating a higher proportion of B cells expressing only the truncated DOCK8. Interestingly, patient CD19^+^ B cells immortalized with EBV expressed only mutated *DOCK8* transcripts (Fig. 2E) suggesting selective outgrowth of cells that did not undergo somatic reversion. As migration of DOCK8-deficient B cells has previously been shown to be impaired [5], we investigated the functionality of the truncated DOCK8 protein in a transwell assay using the patient EBV B cells expressing only the truncated version of DOCK8. Migration of patient EBV B cells was comparable to the EBV B cell lines of healthy controls and significantly better than that of EBV B cells of a patient with complete loss-of-function mutation in DOCK8 (*DOCK8*^null^) (Fig. 2F). This shows that migration was not significantly affected by the truncation of the DOCK8 protein.

DOCK8 is a large protein with at least two described functional domains, the N-terminal DOCK homology region (DHR) 1 domain and the C-terminal DHR2 domain. There is only little data available on which downstream cellular functions are mediated by each domain. Our data showing normal migration of patient EBV cells expressing a truncated DOCK8 protein in which the C-terminal DHR2 domain is disrupted suggest that this domain is dispensable for lymphocyte migration. In line, Ham et al. [6] showed that the N-terminal region of DOCK8, but not the C-terminal region of DOCK8 is a crucial binding site for Wiskott–Aldrich syndrome protein (WASp), a protein involved in actin cytoskeleton remodeling and migration.

The C-terminal DHR2 domain of DOCK8 exhibits the guanine nucleotide exchange in the small GTPase cell division control protein 42 homolog (CDC42). To our knowledge it is not yet clear which cellular functions are modulated by signaling events initiated by the DOCK8-mediated guanine nucleotide exchange. Absent T cell proliferation when the patient presented initially in clinic, presumably before somatic reversion took place, suggests that lymphocyte proliferation is likely modulated by the guanine nucleotide exchange function in the DHR2 domain. Further in-depth characterization of truncated DOCK8 proteins will provide a valuable tool for gaining a better understanding of the function of DOCK8 in immune regulation and genotype–phenotype correlations in various patients with DOCK8 deficiencies.

Currently, curative hematopoietic stem cell transplantation (HSCT) is the definitive treatment for DOCK8 deficiency. Without HSCT, infections and an increased risk of developing malignancies due to impaired clearance of oncogenic viruses are life-threatening complications associated with DOCK8 deficiency. HSCT is expected to prevent both; however there is no data available on the development of malignancies post-HSCT. In our patient, expression of truncated, partially functional DOCK8 in combination with somatic reversion in T cells is sufficient to maintain antiviral immunity, as shown by the absence of severe viral infections. Therefore, the risk versus benefit of HSCT is unclear in patients with less severe disease and demands careful consideration.

## 5 Conclusion

Here we reported a patient with atypical DOCK8 deficiency characterized by a much milder phenotype of the immunodeficiency compared to classical DOCK8 deficiency which further broadens the spectrum of DOCK8 associated diseases. As suggested by normal patient EBV B cell migration and initially absent T cell proliferation in addition to somatic reversion of *DOCK8* predominantly in T cells, this relatively mild phenotype is the result of hypomorphic DOCK8 function and somatic reversion.

## Conflict of interest statement

None of the authors has any potential financial conflict of interest related to this manuscript.

## Funding

This work was supported by the Oxford Partnership Comprehensive Biomedical Research Centre with funding from the Department of Health's National Institute of Health Research (NIHR) (IS-BRC-0211-10025) Biomedical Research Centre funding scheme. The views expressed in this publication are those of the authors and not necessarily those of the Department of Health. B.F. was supported by the NIHR Chief Scientist Funding.

## Figures and Tables

**Table 1:** Immunological characteristics of the patient.

Parameter	Patient		Normal range
*Serum immunoglobulin*			
IgM (g/L)	↓	0.23	0.4–2.5
IgG (g/L)	N/(↓)^a^	7.04	6.0–13.0
IgA (g/L)	N	2.75	0.8–3.0
IgE^b^ (kU/L)	N	190	< 380

*Leukocyte count (no./μL) and phenotype (%)*			
Lymphocytes (/μL)	N	1170	1000–5300
CD19^+^ B cells			
CD19^+^ (/μL)	N	530	200–600
CD38^++^ IgM^++^ transitional (%)	↑	14.2	4.6–8.3^c^
CD27^−^ IgD^+^ naive (%)	↑	91	47.3–77.0^c^
CD27^+^ IgD^+^ natural effector (%)	↓	3.44	5.2–20.4^c^
CD27^+^ IgD^−^ switched memory (%)	↓	1.12	10.9–30.4^c^
CD3^+^ T cells			
CD3^+^ (/μL)	↓	570	800–3500
CD4^+^ (/μL)	↓	180	400–2100
CD4^+^ CD45RA^+^ naïve (%)	↓	39	46–77^d^
CD4^+^ CD45RO^+^ memory (%)	↑	61.1	13–30^d^
CD4^+^ CD45RA^+^ CD31^+^ RTE (%)	↓	32.5	42–74^d^
CD8^+^ (/μL)	N	280	200–1200
CD8^+^ CD45RA^+^ naïve (%)	↓	41.4	63–92^d^
CD8^+^ CD45RO^+^ memory (%)	↑	58.6	4–21^d^
CD16^+^ CD56^+^ NK cells (/μL)	N	80	70–1200
Eosinophils (/μL)	↑	1150	< 350
TRECs (/10^6^ MNC)	↓	1197	> 10,000
Specific IgG responses			
Tetanus toxoid (IU/ml)	N	0.03	> 0.01
*Haemophilus* influenzae type b (μg/ml)	↓	< 0.15	0.15–1.0
Pneumococcal polysaccharides (U/ml)	↓	1	> 14
Measles	Absent		
Varicella zoster	Absent		
T cell proliferation			
PHA	↓↓ absent at 10 years of age ↓ decreased at 15 years of age		

N, value within normal range; ↑, value above normal range; ↓, value below normal range; RTE, recent thymic emigrants; TREC, T cell receptor rearrangement excision circle; PHA, phytohemagglutinin.

a Serum IgG dropped within a year after initial presentation.

b Serum IgE was measured after identification of the DOCK8 mutation on serum samples frozen before start of immunoglobulin replacement therapy.

c 5–95 percentile range for age-matched controls adopted from [7].

d 10–90 percentile range for age-matched controls adopted from [8].
