# Supplementary material for: Hypomorphic function and somatic reversion of DOCK8 cause combined immunodeficiency without hyper-IgE
Source: Clin Immunol. 2016 Feb;163:17–21. doi: 10.1016/j.clim.2015.12.003 (PMC4758821; doi:10.1016/j.clim.2015.12.003)
Supplement: Supplementary file 1 — Supplementary material [file mmc1.docx]

**SUPPLEMENTARY DATA**

**Hypomorphic function and somatic reversion of DOCK8 cause combined immunodeficiency without hyper‑IgE**

**Anne-Kathrin Kienzler,^1^ Pauline A van Schouwenburg,^1^ John Taylor,^2^ Ishita Marwah,^1^ Richa U Sharma,^1^ Charlotte Noakes,^2^ Kate Thomson,^2^ Ross Sadler,^3^ Shelley Segal,^4^ Berne Ferry,^3^ Jenny C Taylor,^5^ Edward Blair,^6^ Helen Chapel,^1^ Smita Y Patel,^1^**

^1^Nuffield Department of Medicine, Experimental Medicine Division, University of Oxford, UK and Oxford NIHR Biomedical Research Centre, John Radcliffe Hospital, Oxford, UK

^2^Oxford NHS Regional Molecular Genetics Laboratory, Oxford University Hospitals NHS Trust, Oxford, UK

^3^Department of Clinical Laboratory Immunology, Churchill Hospital, Oxford University Hospitals NHS Trust, Oxford, UK

^4^Department of Paediatrics, Children‘s Hospital, Oxford University NHS Hospitals Trust, Oxford, UK

^5^Oxford Biomedical Research Centre, Wellcome Trust Centre for Human Genetics, Oxford, UK

^6^Department of Clinical Genetics, Churchill Hospital, Oxford University NHS Hospitals Trust, Oxford, UK

**Corresponding author:**

Anne-Kathrin Kienzler, PhD

University of Oxford

NDM Experimental Medicine and BRC Translational Immunology Laboratory

John Radcliffe Hospital, Level 7, Room 7400

Headley Way

Oxford, OX3 9DU, UK

Email: anne-kathrin.kienzler@ndm.ox.ac.uk

Phone: +44 (0) 1865 857620

**METHODS**

**Study approval**

Anticoagulated peripheral blood samples were obtained from the patient, her parents and healthy individuals after the provision of informed, written consent.

The patient and parents gave written consent (12/SC/0044) and the studies were performed according to the Declaration of Helsinki.

The parent-child trio were consented for exome sequencing; however, their consent was restricted to genes related to their condition and not for whole exome sequence (WES) analysis. Consequently, the patients have not consented for all data generated by WES or array CGH to be publically available. Therefore, we are not able to provide WES data beyond the immune-related targeted analysis.

**DNA isolation**

DNA extraction was performed using the FlexiGene DNA kit (Qiagen) according to the manufacturer’s instructions.

**Flow cytometry**

Immunophenotypes were determined using whole blood and standard protocols with mixtures of fluorochrome-conjugated mAbs and absolute count beads (TruCount, BD Bioscience) according to manufacturer’s instructions. To monitor lymphocyte proliferation, PBMCs were labeled with CFSE (carboxyfluorescein diacetate, succinimidyl ester; Molecular Probes) according to manufacturer’s instructions. After six days, CFSE dilution and T cell phenotype was determined by flow cytometry with following antibodies: CD3 PerCP (SK7), CD3 Alexa Flour 700 (SK7), CD4 APC (SK3), CD4 Qdot605 (S3.5), CD8 Pacific Blue (3B5). Dead cell exclusion was performed with LIVE/DEAD® Fixable Aqua Dead Cell Stain Kit (life technologies).

**Exome sequencing and analysis**

Exome capture was performed using the NimbleGen SeqCap EZ Human Exome Library v2.0, according to the manufactures instructions, and sequenced using a 100 bp paired-end read protocol on an Illumina HiSeq. Approximately 15Gb of sequence were obtained for each of the three individuals (patient and parents), providing at least 10x vertical read depth over ~90% of the coding exome, as specified by the consensus coding sequence (CCDS) project. Reads were aligned to hg19 with Stampy (v1.0.20) ([1](#_ENREF_1)) and variant calling of single nucleotide variants (SNVs) and short insertion and deletions (indels) was undertaken using Platypus (v0.5.2). ([2](#_ENREF_2)) The variants were annotated and restricted to 200 immune related genes (IUIS list of PIDs ([3](#_ENREF_3))), using the Illumina VariantStudio data analysis software. Variants were initially filtered on a population frequency below 5% within the NHLBI GO Exome Sequencing Project (<http://evs.gs.washington.edu/EVS/>), which identified a single nucleotide duplication within *DOCK8*. Further scrutiny of SNVs within this gene identified two upstream SNPs (rs506121 and rs529208) that were homozygous in the patient and inherited from the mother who was heterozygous. A comparison of the mapped *DOCK8* exonic read count for each individual suggested a paternally inherited deletion was present within the 5’region of this gene.

**Genomic DNA sequencing**

M13-tagged oligonucleotide primers targeting exon 47 of *DOCK8* (GenBank accession number: NM_203447.3) were used to amplify genomic DNA (Forward 5’-GACCACTGGAAGTAGCCCA-3’; Reverse 5’-TGCACTTTGAGAACCACTGC-3’). PCR products were amplified using Kappa 2G FAST DNA Polymerase (KAPA biosystems), according to the manufacturer’s instructions, and purified using the Agencourt Ampure system (Beckman Coulter). Dideoxy Sanger sequencing was undertaken using universal M13 primers, BigDye Terminator kit 3.1 (Applied Biosystems), and purified using the Agencourt CleanSEQ system. Capillary electrophoresis was performed using an ABI Prism 3730 Genetic Analyser. cDNA numbering uses A for the initiating ATG as nucleotide 1 and the initiating ATG as codon 1. Sequence conservation of the DHR2 domain was analyzed in mutationtaster (www.mutationtaster.org).

**Microarray-based comparative genomic hybridization (CGH)**

Array CGH was performed on genomic DNA using the Agilent ISCA 8x60K array according to the manufacturer’s protocol and scanned using an Agilent Surescan high resolution scanner. The position of the array targets was mapped to the UCSC genome browser release February 2009 (GRCh37/hg19). Data was analyzed using Agilent CytoGenomics 2.0 software package (genome build hg19) and restricted to the *DOCK8* locus on chromosome 9. The absence of upstream probes prevented an accurate size estimate of the deletion (9p24.3 (204,193-343,954)), which encompasses exons 1-14 of *DOCK8*.

**T cell proliferation**

To analyze T cell proliferation 1x10^6^ peripheral blood mononuclear cells (PBMCs)/ml were stimulated for six days with 2.5 µg/ml phytohaemagglutinin (PHA; Sigma-Aldrich) in RPMI-1640 medium supplemented with 10% FCS, 10 U/ml penicillin, 10 µg/ml streptomycin, 1 mM sodium pyruvate, 1% non-essential amino acids, 2 mM L‑glutamine and 50 µM β-mercaptoethanol.

**Generation of Epstein-Barr Virus (EBV)–immortalized B cell lines**

Magnetic bead sorted CD19+ B cells (B cell isolation kit II, human, Miltenyi Biotec) were immortalized with Epstein-Barr-Virus-containing supernatant according to standard protocols.

**Western Blot**

Cell lysates were prepared using lysis buffer containing 50 mM Tris-HCl pH 8, 150 mM NaCl, 1 % NP‑40 and Complete Protease Inhibitor (Roche). Incubation with antibodies specific for DOCK8 (H159, Santa Cruz Biotechnology) and β-actin (AC15, Sigma-Aldrich) was performed in PBS / 5 % non-fat milk / 0.1 % tween, followed by incubation with respective HRPO-coupled antibodies (Jackson Immunoresearch or Sigma-Aldrich). Finally, membranes were incubated in SuperSignal West Pico Chemiluminescent Substrate (Thermo Scientific).

**RNA extraction, reverse transcription, DOCK8-specific PCR and Sanger sequencing**

RNA was extracted using TRIZOL reagent (Invitrogen) and quantified using the NanoDrop 1000 (Thermo Scientifc). cDNA synthesis was performed with equal amount of RNA using SuperScript III reverse transcriptase (Invitrogen) and random hexamer primers (Amersham Pharmacia Biotech) according to standard protocols. DOCK8-specific PCR (transcript ID: ENST00000453981) was performed using Paq DNA polymerase (Agilent Technologies), and the following primers and amplification program: Forward 5’‑CATGAGCAGTACAGAAGGAACA‑3’ and reverse 5’‑ACTGGGTTTCACATTTCCTGAA‑3’, and 3 min at 95°C followed by 32 cycles consisting of 30 sec at 95°C, 30 sec at 58°C, and 45 sec at 72°C. After amplification, the DOCK8-specific PCR product was cleaned up using QIAquick PCR purification kit (Qiagen) and subjected to standard Sanger sequencing using DOCK8-specific primers listed beforehand. Sanger sequencing traces were visualized in Chromas (Technelysium Pty Ltd).

**Peak height quantification in sequencing traces**

To determine the percentage of wild-type and mutated *DOCK8* transcripts in respective cDNA samples, Sanger sequencing traces were subjected to quantitative analysis using the ab1 Peak Reporter tool (http://apps.lifetechnologies.com/ab1peakreporter). Ab1 sequencing files were converted into numerical peak height data of base traces by applying the ‘ratio of maximal signals in a 7-scan window’ calculation provided by the software. Subsequently, numerical peak height data were analyzed in Microsoft Excel. Percentages of wild-type and mutated *DOCK8* transcript sequences are depicted as the mean of the sequence contributing nucleotide percentages of the analyzed 20 nucleotide positions. Non wild-type or non-mutated bases are called at a rate of about 5% which gives a total sequencing base call background of 10%.

**Pyrosequencing**

The c.6019dupT variant was detected in the separated cell population by pyrosequencing. PCR templates for pyrosequencing were amplified from cDNA (10 ng) using the biotinylated forward primer 5' TCCTGCTGATCCAAAACT 3' and reverse primer 5' GCCATTTTCCTTCTTACC 3'. Following PCR amplification, the biotinylated PCR products were placed in 96-well plates and bound to streptavidin-coated sepharose beads (GE Healthcare, Piscataway, NJ, USA). The PCR products were denatured, and the non-biotinylated fragments were washed from the beads using the Pyromark Q96 Vacuum Workstation (Qiagen). The beads were then resuspended in annealing buffer (40µL) containing 0.4 pmol of the sequencing primer (5' TCAACTTGTTGTGATGTCG 3'). Pyrosequencing was performed using the Pyro Gold Q96 reagents (Qiagen), using dispensations based on the target sequence with the Pyromark Q96 system. The PCR templates and pyrosequencing reactions were performed in triplicate. Raw data files were imported into excel for ratio calculations. “T” nucleotide phosphorescence signal ratios were calculated by dividing the phosphorescence signal of the “T” nucleotide at position c.6018‑19 by the phosphorescence signals at 1 and 2 nucleotide positions up- and downstream of c.6018‑19 (T/-1, T/+1, T/-2, T/+2). A reduced “T” nucleotide signal ratio indicates a lower mutational load which is equal to an increased rate of somatic reversion.

**Transwell migration assay**

Before transwell migration assays, EBV-transformed B cells were starved overnight in migration medium (RPMI‑1640 supplemented with 0.25% fatty acid‑free BSA (Sigma-Aldrich)). On the day of experiment, EBV cells (100 μL of 2 × 10^6^ cells/mL) in migration medium were added to the upper chamber of a Transwell (8 µm pore size; Millipore). The lower chamber contained 600 μL of migration medium enriched with 10% FCS. After 4 hours of migration at 37°C, cells from the lower chamber were collected, stained with DAPI (4',6-diamidino-2-phenylindole) for dead cell exclusion, and recorded on a flow cytometer (BD Fortessa) by timed acquisition. Specific migration was calculated as follows:

$$specific migration =\left[ \frac{\# migrated cells\times100}{\# input cells} \right] -\left[ migration at 0\% attractant \right]$$

**Statistics**

Analyses were performed with PRISM software (GraphPad Software, Inc.). Statistical hypotheses were tested using the unpaired 2-tailed t test. Differences were considered significant if the P value was less than 0.05.

**REFERENCES**

1. Lunter, G., and Goodson, M. 2011. Stampy: a statistical algorithm for sensitive and fast mapping of Illumina sequence reads. *Genome Res* 21:936-939.

2. Rimmer, A., Phan, H., Mathieson, I., Iqbal, Z., Twigg, S.R., Consortium, W.G.S., Wilkie, A.O., McVean, G., and Lunter, G. 2014. Integrating mapping-, assembly- and haplotype-based approaches for calling variants in clinical sequencing applications. *Nat Genet* 46:912-918.

3. Al-Herz, W., Bousfiha, A., Casanova, J.L., Chapel, H., Conley, M.E., Cunningham-Rundles, C., Etzioni, A., Fischer, A., Franco, J.L., Geha, R.S., et al. 2011. Primary immunodeficiency diseases: an update on the classification from the international union of immunological societies expert committee for primary immunodeficiency. *Front Immunol* 2:54.
